# Comparative Assessment of the Periodontal Findings in Child Subjects With a Normal Body Mass Index and in Obese Subjects

**DOI:** 10.7759/cureus.47897

**Published:** 2023-10-29

**Authors:** Sneha Suresh, Abhishek Anand, Pinky Singh, Niharika Shahi, Swati Sharma, Ankur Jethlia

**Affiliations:** 1 Department of Periodontology and Implantology, Buddha Dental College and Hospital, Patna, IND; 2 Department of Pedodontics and Preventive Dentistry, Netaji Subhash Medical College and Hospital, Patna, IND; 3 Department of Conservative and Endodontics, Dr. B. R. Institute of Dental Sciences and Hospital, Patna, IND; 4 Department of Pedodontics and Preventive Dentistry, Purvanchal Institute of Dental Sciences, Gorakhpur, IND; 5 Department of Pedodontics and Preventive Dentistry, Dental Institute Rajendra Institute of Medical Sciences (RIMS), Ranchi, IND; 6 Department of Maxillofacial Surgery and Diagnostic Sciences, Diagnostic Division, College of Dentistry, Jazan University, Jazan, SAU

**Keywords:** probing depth (pd), periodontitis, obesity, gingivitis, clinical attachment loss (cal), body mass index (bmi), bleeding on probing (bop)

## Abstract

Background: Obesity in children is a concerning issue affecting a large population globally. Obesity and overweight are risk factors for various medical conditions, including periodontal diseases, hypertension, cerebrovascular disease, cardiovascular disease, and/or diabetes.

Aim: The study aimed to comparatively assess the periodontal findings in child subjects with a normal BMI and in obese subjects.

Methods: The present observational study aimed to comparatively assess 216 school-going child subjects that were divided into two groups: non-obese (BMI<25) and obese, with BMI≥25 having equal gender distribution. In both groups, clinical attachment loss (CAL), probing depth (PD), and bleeding on probing (BOP) were assessed along with a questionnaire on oral hygiene and dietary habits. The data gathered were statistically analysed.

Results: The study results showed that in obese subjects, significantly higher values were seen for probing depth, bleeding on probing, and plaque index compared to non-obese subjects with p<0.05. However, no significant difference was noted in the CAL of obese and non-obese subjects (p>0.05).

Conclusion: The periodontal status is compromised in obese subjects with higher values of probing depth, bleeding on probing, and plaque index compared to child subjects with normal weight. The level of CAL does not differ significantly between obese and non-obese child subjects.

## Introduction

Obesity is increasing globally in developing and developed countries and is defined as increased body weight concerning body mass with excessive fat that affects health negatively. Obesity is a multifactorial and chronic condition arising from socioeconomic, cultural, environmental, behavioural, metabolic, or genetic conditions [1].

Obesity is a condition in children arising secondary to changes in dietary habits and lifestyle, with increasing prevalence globally, including in India. A cross-sectional school-based study was conducted among 1185 secondary school students in Udupi, India [2]. They found that 7.1% of males, 5.4% of females, and an average of 6.2% of students have obesity. Obesity is a major condition that causes hypertension, type II diabetes, pulmonary conditions, and cardiovascular diseases. Obesity decreases the quality of life in subjects with low socioeconomic status and young adults [3]. BMI is the most commonly used index to measure body fat and represents body mass in kilogrammes divided by body height in metres squared [3].

Obesity develops a proinflammatory state systemically by exerting immunologic and metabolic body effects, increasing the susceptibility to a chronic infectious disease, periodontitis. The correlation between periodontitis and obesity can be understood through cytokines and hormones from the adipose tissues, known together as adipokines [3]. Increased adipokines from visceral fat molecules cause clogging with blood cells, which forms microemboli in the microvasculature, reducing blood supply to the gingiva and increasing periodontal disease progression [3].

According to the Global Burden of Disease Study (2016), severe periodontal disease was the 11th most prevalent condition in the world [3]. The prevalence of periodontal disease was reported to range from 20% to 50% around the world. During the period from 1990 to 2010, there was a 57.3% increase in the global burden of periodontal disease. Adults in Belarus (76%), Germany (73%), and Nepal (64%) demonstrated the highest prevalence of periodontitis. More than half of the adult population in Poland (62%), Malaysia (60%), Libya (56%), Iran (53%), and Taiwan (53%) had periodontitis [4].

Nazir et al. [4] in 2020 observed that for periodontal diseases, the projection is alarming, with the prevalence at present being 45% for the 15+ age group and the actual prevalence in lakhs at 3413.8 (year 2010) and 3624.8 (year 2015).

Adipose tissues secrete tumour necrosis factor-α (TNF-α) that mediates tissue injuries in various organs via endotoxins, including periodontal tissues, which increases periodontal degradation. Previous literature showed a correlation between BMI>40 and TNF-α levels in gingival crevicular fluid (GCF), suggesting obesity as a low-grade systemic inflammatory condition [5].

With the high prevalence of obesity and its association with periodontal disease, the present study aimed to comparatively assess the periodontal findings in child subjects with a normal BMI and obese subjects.

## Materials and methods

The present observational study aimed to comparatively assess the periodontal findings in child subjects with a normal BMI and in obese subjects. The study was conducted at Buddha Institute of Dental Science and Hospital, Patna, Bihar [ECI/BIDSP/103/2022] after clearance was given by the concerned ethical committee. Informed consent was obtained from the parents of all subjects.

The study included 216 school-going child subjects that were divided into two groups (n = 108): non-obese (BMI<25) and obese, with BMI≥25 having an equal gender distribution and age range of 10-12 years.

The exclusions were subjects with menarche (females), as it can affect the periodontal state, non-restorable teeth, unerupted permanent teeth, fixed orthodontic appliances, antibiotics, and anti-inflammatory drugs. To record BMI, height in metres and weight in kilogrammes were recorded. To assess the periodontal status and indices, a clinical examination was done using a periodontal probe (UNC-15 probe) for clinical attachment loss (CAL), probing depth (PD), bleeding on probing (BOP), and PI.

Periodontal Index (PI) = the number of stained surfaces ÷ the total number of tooth surfaces × 100

Clinical assessment of six teeth (all first molars and maxillary right and mandibular left central incisors) is completed with a specially designed periodontal probe (PIT probe with markings at 4, 6, 8, and 11 mm and a 0.5 mm ball tip).

The teeth evaluated were mandibular left central incisors, mandibular right first molars, mandibular left first molars, maxillary right central incisor, maxillary left first molars, and maxillary right first molars. If any of these teeth are missing, the teeth distal or mesial to the missing teeth were selected.

The O'Leary plaque index of 1972 [6], bleeding on probing by van der Velden in 1980 [7], probing depth by Lenox and Kopczyk [8] in 1973, and clinical attachment level were recorded following the study by Sivertson and Burgett [9] in 1976. This was followed by filling out the questionnaire on hygiene and dietary habits. The questions were on toothbrushing frequency in 24 hours, gingival bleeding on brushing, use of fluoridated mouthwashes, number of meals in 24 hours, and use of dental floss. The questionnaire was based on a preformed structured pattern.

All subjects were randomly examined by multiple operators. The principal operator provided and gave training for the examination to co-operators. Data for all subjects were recorded after questionnaire filling, and recordings were made by doing the comprehensive intraoral examination under adequate visibility and illumination.

The data were statistically analysed using SPSS software version 21.0 (IBM, Chicago, USA) along with a t-test and a chi-square test. The results were expressed as means and standard deviations, frequency, and percentage. Significance levels were signified as p<0.05.

## Results

The present retrospective clinical study assessed 216 school-going child subjects that were divided into two groups: non-obese (BMI<25) and obese (BMI≥25). Both groups had an equal gender distribution of 54 males and 54 females. The BMI in the obese group was 27.57±2.22 kg/m^2^, whereas in the non-obese group, the BMI was significantly lower at 18.42±2.07 kg/m^2^ with a p-value of 0.001 as shown in Table 1.

**Table 1:** Body mass index in the two groups of study subjects SD: standard deviation

BMI (kg/m^2^)	Mean± SD	p-value
Obese	27.57±2.22	0.001
Non-obese	18.42±2.07	

Periodontal examination in two groups showed that the mean BOP was significantly higher in obese subjects with 86 compared to non-obese subjects with 24.3 (p<0.05). Similar results were seen for PD and PI with p<0.05 (Table 2). However, the CAL had a non-significant difference between the two groups, where no CAL was seen in the non-obese group and a CAL of 1 and 3 mm was noted in two obese subjects (p>0.05).

**Table 2:** Comparison of periodontal status in two study groups

Periodontal status	Study groups	Mean values and percentages (%)	p-value
Bleeding on probing (%)	Obese	86%	<0.01
	Non-obese	24.3	
Pocket depth (mm)	Obese	2.27	0.04
	Non-obese	1.52	
Plaque index (%)	Obese	97%	0.02
	Non-obese	85	
CAL	Obese	2	<0.001
	Non-obese	0	

On assessing the dietary and food habits of the two groups of study subjects, it was seen that gingival bleeding during brushing was significantly higher in obese subjects compared to non-obese subjects (p<0.05). The use of non-fluoridated mouthwash had a non-significant difference between the two groups (p>0.05). The use of floss was significantly higher in the non-obese group, with 28 subjects flossing regularly compared to the obese group, where only six subjects flossed regularly (p<0.05). The meal frequency was significantly higher in obese subjects compared to non-obese subjects (p<0.05). The frequency of tooth brushing was significantly lower in obese subjects compared to the non-obese group with p<0.05, as depicted in Table 3.

**Table 3:** Oral hygiene and dietary habits in two groups of study subjects n = number of subjects

Questions	Obese n=108 (%)	Non-obese n=108 (%)	p-value
Gingival bleeds during brushing			
Yes	80	16	<0.05
No	28	92	
Fluoridated mouthwash			
Yes	40	58	>0.05
No	68	50	
Floss			
Yes	6	28	<0.05
No	102	80	
Meals			
½	4	30	<0.05
3	26	58	
>3	78	20	
Brushing			
None	80	34	<0.05
Once	22	50	
Twice	6	14	
3/more	-	10	

## Discussion

Obesity in children of Indian origin is concerning, with lots of literature data confined to particular geographic areas and the persistence of obesity into adulthood, leading to systemic diseases. There exists a reciprocal relationship between dental health and diet. Daily eating habits have an impact on oral health; obesity engages in a systemic, generalised inflammatory process, influencing metabolic markers and raising the risk of periodontal conditions.

As observed by Chadda and Sengupta [10] in 2002, an estimated 20 million youngsters between the ages of 10 and 14 are dependent on tobacco, as per a survey conducted by the National Sample Survey Organisation by the Indian Government. About 5500 new users are added to this incredible amount every day, for a total of two million new users annually. This was also a major cause of the increasing number of periodontal diseases in children [10].

Among African populations, conditions of oral hygiene are generally deficient, and the methods used for oral cleaning can be unconventional (such as using sticks or sponges with gum, banana stems with carbon powder, or plant leaves with ash used with cotton, cloth, or fingers) and lead to plaque formation and consequently calculus formation, even at early ages, as reported by Nasreen et al. [11] in 2021. Oral health and diet share a correlation suggested by Tramini et al. [12] in 2009. Obesity leads to a generalised inflammatory body state, increasing the risk of periodontal disease, as reported by Van Dyke [13] in 2008 and Reeves et al. [14] in 2006.

The study results showed a significantly higher PI in obese children compared to non-obese children (p<0.05). These results were consistent with Sfasciotti et al. [15] in 2016 and Scorzetti et al. [16] in 2013, where authors reported higher PI in obese subjects, which can be attributed to poor oral hygiene, carbohydrate-rich diets, and higher meal frequency. These results contrasted with Zuza et al. [17] in 2017, where a small sample size was considered.

BOP was higher in obese subjects (p<0.05) than in non-obese children. These results are confined to Scorzetti et al. [16] in 2013 and Zuza et al. [17] in 2017, which can be due to a rise in levels of pro-inflammatory cytokines in the GCF of obese subjects reported by Modeer et al. [18] in 2011. In subjects with high BMI, an increase in leptin, TNF-α, and IL-6 is seen, which can cause inflammation and destruction of the periodontium, as suggested by Modeer et al. [18] in 2011.

The study results reported significantly higher mean PD in obese subjects compared to non-obese subjects. These results were in agreement with Scorzetti [16] in 2013, where more pockets were prevalent in obese subjects, and with Zuza et al. [17] in 2017, where periodontal status was better in subjects with a normal BMI.

The questionnaire showed less attentive oral hygiene in obese subjects, where the relationship was considered metabolic and immunologic, causing periodontitis. This is also attributed to different salivary compositions in obese subjects with oral microflora predisposing to obesity and periodontitis, as reported by Di Baise et al. [19] in 2008.

Limitations

The study had limitations because no education was provided to the subjects prior to the procedure. The subjects from both groups should have been given similar instructions and then must be included. Subjects with similar plaque indexes would have provided better results concerning the relationship between periodontal indices and BMI, eliminating the confounding factors. The study also had the limitation of including subjects from different socioeconomic backgrounds, which can affect periodontal status.

## Conclusions

The periodontal status is compromised in obese subjects with higher values of probing depth, bleeding on probing, and plaque index compared to child subjects with normal weight. The level of CAL does not differ significantly between obese and non-obese child subjects. The study suggests promoting oral health knowledge in obese subjects and further research.

## Appendices

Questionnaire for each patient included in the study

[A]

1. Name:

2. Age:

3. Gender:

4. Address:

5. Marital Status:

[B]

1. How many times do you brush your teeth everyday

a)      None

b)      Once

c)      Twice

d)     Thrice or more

2. Gingival bleeds during brushing

a)      Yes

b)      No

2. Use of fluoridated mouthwash

a)      Yes

b)      No

3. Flossing habit

a)      Yes

b)      No

4. How many times do you take meal

a)      Once or twice a day

b)      3 times a day

c)      >3 times a day
